# Supplementary material for: Development of a questionnaire to determine the case detection delay of leprosy: A mixed-methods cultural validation study
Source: PLoS Negl Trop Dis. 2022 Jan 13;16(1):e0010038. doi: 10.1371/journal.pntd.0010038 (PMC8758092; doi:10.1371/journal.pntd.0010038)
Supplement: Attachment — Submitted filename: Response to reviewers CDD tool manuscript.docx [file pntd.0010038.s002.docx]

Dear editors and reviewers,

We hope this letter finds you in good health.

Via this rebuttal letter, we wish to thank you for your valuable comments on our manuscript. It resulted in new insights and valuable adjustments to the manuscript. In the table below, we have included our responses to the comments you have shared, accompanied by snippets of the manuscript that were adapted and the according line numbers. We hope this provides you with the information required.

Do not hesitate to contact us if more information is needed.

Again, thank you for your valuable reflections.

Kind regards,

Naomi de Bruijne, Kedir Urgesa, Abraham Aseffa, Kidist Bobosha, Anne Schoenmakers, Robin van Wijk, Thomas Hambridge, Mitzi Waltz, Christa Kasang and Liesbeth Mieras

| Manuscript section | Reviewers’ comment | Authors’ reaction | Line numbers | Changes made to manuscript (highlighted) |
| --- | --- | --- | --- | --- |
| Title | Mixed methods include cross sectional |  | 1 – 2 | Development of a questionnaire to determine the case detection delay of leprosy: A mixed-methods cultural validation study |
| Abstract | Why this conclusion here? | This sentence is not meant as a conclusion but rather to provide background information about the project being part of a large multi-country project. |  |  |
| Abstract | It would be useful if methods section is somewhat in these lines: Reviews of existing CDD questionnaires in related areas further contributed to questionnaire development and together helped to generate a large pool of items which was then subjected to...............scaling method for selection of items from this pool. Face and content validity were obtained, following which internal consistency and test, re-test reliability were assessed. Scaling exercise resulted in XX items being discarded from an initial pool of XXX, owing to the poor agreement among experts regarding relevance. Face and content validity were good with experts endorsing relevance and applicability of items. The intra-class correlation coefficient (ICC) for test re-test reliability of the XX item scale was ... (95% CI: .....) indicating marginal intra-class correlation. The overall Cronbach’s alpha was XX while the alphas for each of the components was.... | Thank you for your extended comment. We wish to clarify that there were no standard tools available to measure the case detection delay in this context. The current tool is therefore designed based on questionnaires and interview guides other researchers developed, used or tried out in the field. These outcomes were combined with expert opinion to design the current tool (see further Figure 2 in manuscript).  Furthermore, the comment formulated proposes to include Crohnbachs alpha in the methodology and result section. As the current tool is a questionnaire, not a scale, that is designed to get an estimate of the case detection delay, we consider the Crohnbachs alpha not relevant in this context. We hope to have clarified this, however, if any questions or unclarities arise, we are available to discuss this further. |  |  |
| Abstract | Delay is caused not only by patient but health provider too | Thank you for this comment. We agree with your statement, however, this section is about determining the full case detection delay rather than the reasons behind the case detection delay. In the manuscript, under Item relevance (line number 282), the health system delay is discussed and taken into account in the tool. |  |  |
| Abstract | Primary focus is to check is questionnaire is valid | We agree with this remark, thank you. | 53-56 | Although the instrument has promising potential for use in future leprosy research, it is recommended that the tool is further validated, also in other regions or countries, to ensure cultural validity and to examine psychometric properties like test-retest reliability and interrater reliability. |
| Introduction | This content of sign & symptom should be captured in the tool | This distinction has been taken into account in the tool and is integrated accordingly. We clarified the sentence in the article a bit. | 112-113 | For reasons of readability of the current article, when mentioned ‘signs’ in the further text this should be read as ‘signs and symptoms’. |
| Introduction | May be rephrased | Thank you, we have done accordingly. | 136-138 | To assess the CDD of leprosy in these three East African countries (Figure 1), it is necessary to design a methodological tool that is validated and adapted to the cultural context of these countries (19). |
| Introduction | obj is to develop a validated, culturally appropriate questionnaire to measure ..... in..... | Thank you for your suggestion, we have adjusted the sentence accordingly. | 143-145 | Therefore, the objective of this study was to design a methodological tool ~~that can be used~~ to determine the delay in diagnosis of leprosy in number of months, that is validated for usage in Ethiopia, Mozambique and Tanzania, and adaptable to other cultural settings. |
| Methods | More details needed on validation process, reliability assessments | Thank you for the comment. We wish to repeat that the current tool designed is a questionnaire and not a scale. The details available on validation in this study are shared in the methods and results sections of the manuscript. We agree that further (cultural) validation of the tool is needed before use in other settings, as is described in the discussion.  Furthermore, we wish to share that further validation of the questionnaire is done in local studies which will be published in follow-up of the current article. These studies will address measurement consistency, including interrater reliability and test-retest reliability. However, in our opinion, internal consistency is not applicable for this questionnaire. | 439-441 | Considering the low number of recently diagnosed leprosy patients per health center, a prolonged field study is necessary to assess the measurement consistency (i.e. test-retest reliability and inter-rater reliability) of the questionnaire. |
| Methods | Any sampling done? | Yes, thank you for addressing this. A table was added that includes the sampling strategies used per sample. | 184 | *A table was added, including inclusion criteria, exclusion criteria and the sampling strategies per sample.* |
| Methods | Suggestion to add appropriate key words. | Thank you for this suggestion. The key words were added to the title page. | 19-20 | Key words: leprosy, case detection delay, early detection, NTD, SDR-PEP, mixed methods, Ethiopia, Mozambique, Tanzania, cultural validation. |
| Methods | Suggestion to add abbreviations. | Thank you for this suggestion. Abbreviations were added to the title page. | 21-26 | Abbreviations: Armauer Hansen Research Institute (AHRI), Case detection delay (CDD), Catholic University of Health and Allied Sciences (CUHAS), German Leprosy and Tuberculosis Relief Association (DAHW), Focus groups discussion (FGD), General practitioner (GP), International Federation of Anti-Leprosy Associations (ILEP), Multidrug therapy (MDT), Post-exposure prophylaxis (PEP), Single dose rifampicin (SDR), Sub-Saharan Africa (SSA), Tuberculosis (TB), World Health Organization (WHO). |
| Methods | When describing the population the inclusion and exclusion criteria might differ for each of the groups included in the study. So I would suggest to elaborate or add a box/table for clarity. | We agreed with your proposal and have added a table including these details. Thank you for addressing this. | 184 | *A table was added, including inclusion criteria, exclusion criteria and the sampling strategies per sample.* |
| Methods | I would suggestion authors to describe the sample size for each category of the persons included in the study: people affected with leprosy, health care workers, key informants and the researchers. | The sample size of the samples included are described in Table 2. | 244 |  |
| Results | this refernce material may be shared | Thank you for this comment. The information collected via literature review will be made publicly available as required. |  |  |
| Results | Table 2 needs correcting(See below in Editorial section) | Thank you, changes were made accordingly | 394 | *One of the top two repeating lines in Table 3 (previous Table 2) was deleted.* |
| Results | Table 2: Lines one and two are the same. Please check. | See above. | 394 | *See above.* |
| Results | Include final standardized version of the questionnaire for the reader with a link to the guidelines for administrating it. | This will be done, thank you. |  |  |
| Results | On the results, it would be interesting to know the grade of disability to correlate with the CDD and could be an indicator of the sensitivity of the questionnaire. | Thank you for your comment and good suggestion. We added a figure with boxplots to show the relation between the case detection delay and the disability grades for the pilot group in Ethiopia. Although ideally, we would have seen longer delays in the dg2 groups compared to the other groups, unfortunately, this was not the case. See further, Figure 3 in the manuscript. | 374-375 | The relation of the CDD of leprosy patients to the disability grade at the moment of diagnosis is shown in the boxplots in Figure 3.  *Figure 3 was added, see line number 382.* |
| Discussion | Why this discussion on reasons for delayed diagnosis, while the core objective of the study is different | We agree with your proposal and have deleted the paragraph accordingly. | 405-406 | ~~In this study, a lack of public knowledge of leprosy was mentioned as the main reason for a delayed diagnosis, which is consistent with other studies, including a recent systematic review by Dharmawan et al. (manuscript submitted) (48–51). The findings in this study regarding patient journeys before diagnosis seem to be consistent with other studies in Ethiopia; Amenu et al. (2000) and Abeje et al. (2016) found the delay of diagnosis to be due to consultation of traditional or religious healers, and the ‘wait and see’ mentality (27,52). Also, misdiagnosis is a common reason for a delayed diagnosis: Abeje et al. (2016) concluded that only 18% of the general health workers in Amhara and Oromia States in Ethiopia was able to correctly diagnose leprosy (52).~~ |
| Editorial and data presentation | Alignment within References needs to be corrected. It changes after 12 on line 522. | Thank you for noting this. | *507* | *Adapted accordingly in manuscript.* |
